# Prevalence and Associated Factors of Adolescent Obesity among Rural School Adolescents in Nepal: A Cross-Sectional Study

**DOI:** 10.1155/2023/2957278

**Published:** 2023-09-29

**Authors:** Deekshanta Sitaula, Aarati Dhakal, Nimesh Lageju, Amisha Silwal, Samjhana Kshetri Basnet, Niki Shrestha, B. C. Anup Bikram, Niraj Phoju

**Affiliations:** ^1^Rasuwa Hospital, Dhunche, Rasuwa, Bagmati Province, Nepal; ^2^Department of Community Programs, Dhulikhel Hospital, Kathmandu University, Kavre, Nepal; ^3^Department of Medical Oncology, Nepal Cancer Hospital and Research Center, Lalitpur, Nepal; ^4^Department of Community Medicine, Chitwan Medical College, Chitwan, Nepal; ^5^NCD and Mental Health Section, Epidemiology and Disease Control Division, Kathmandu, Nepal

## Abstract

**Background:**

Overweight and obesity are major risk factors for chronic diseases and are the leading cause of mortality worldwide. Obesity during adolescence is strongly associated with adulthood obesity leading to increased morbidities and mortality. As a developing country undergoing rapid urbanization, Nepal is in a transitional phase where undernutrition coexists with obesity; however, there is a dearth of literature on the status of adolescent obesity in the rural section of Nepal. The aim of this study was to determine the prevalence of adolescent obesity in a rural district of Nepal and find out its associated factors.

**Methods:**

An institution-based cross-sectional study was conducted among the adolescent students studying in classes 8, 9, and 10 in four secondary schools of Gosaikunda rural municipality of Rasuwa district, Nepal. Total enumerative sampling was used, and Global School-based Health Survey (GSHS) standard questionnaires were used in collecting the information. Height and weight measurements were taken, and the body mass index was classified according to Asia-Pacific criteria. The collected data were analyzed using “SPSS” version 22. Logistic regression analysis was used to evaluate the associated factors, and a *p* value of ≤0.05 was considered statistically significant.

**Results:**

Out of 267 adolescents, 14.6% were obese and 14.6% were overweight. Of the female participants, 39.6% were either obese or overweight, while 12.6% of the male adolescents were overweight/obese. Among the total participants, 16.5% were underweight, including 14.0% females and 20.4% males. Female adolescents were about five times (AOR: 5.2, 95% CI (2.5–10.9), *p* < 0.01) more likely of being overweight/obese than male adolescents.

**Conclusions:**

More than one-quarter of the adolescents were found to be obese/overweight, and the prevalence was significantly more among female adolescents. It emphasizes the necessity of school-based programs promoting a healthy lifestyle among students to maintain healthy weight status.

## 1. Introduction

Overweight and obesity have been ranked as leading causes of mortality worldwide, with over 4 million individuals dying every year as a result of being overweight and obese [1]. There has been a marked rise in the prevalence of overweight and obesity among children, adolescents, and adults around the world in the last few decades [2]. The global age-standardized prevalence of obesity increased from 0.7% in 1975 to 5.6% in 2016 in girls of 5–19 years range, and in boys, it went from 0.9% in 1975 to 7.8% in 2016. Over the course of 40 years (1975–2016), the number of girls with obesity increased by 10-fold, and there was a 12-fold rise in the number of boys with obesity [2]. Overweight and obesity are major risk factors for a variety of chronic diseases including type 2 diabetes, cardiovascular diseases, and cancer [3, 4]. Obesity during childhood and adolescence is strongly associated with obesity in adulthood, leading to increased morbidities and mortality [5]. Adolescents with obesity are also at a higher risk of having obese children later in their life [6]. Childhood and adolescent obesity is associated with genetic factors, socioeconomic status, and behavioral and nutritional factors such as insufficient physical activity, insufficient consumption of fruits/vegetables, inadequate sleep, sedentary activities (watching TV and playing computer games), drinking sugar-containing beverages, eating junk food, smoking, and alcohol consumption [7, 8].

The prevalence of childhood overweight and obesity has increased rapidly in low- and middle-income countries (LMICs) during previous few decades [2, 9, 10]. In a recent study reported from India, the prevalence of obesity among adolescents was 6.8% and about 17.1% of adolescents were overweight [11]. Likewise, in a review study, the prevalence of adolescent obesity and overweight in Asian countries was reported to be 8.6% and 14.6%, respectively [12]. Due et al. in their study reported that the prevalence of overweight was higher among adolescents from less affluent socioeconomic backgrounds in most (21 out of 24) of the western countries [13].

Few studies have been reported from Nepal on childhood obesity, most of which were conducted in urban areas of the country [14–17]. In a study conducted among urban primary school students, the prevalence of childhood overweight and obesity was reported to be 18.6% and 7.1%, respectively [14]. In another study conducted among school students of 6–13 years studying in urban private schools, 14.6% were overweight and 11.3% were obese [15]. The prevalence of adolescent overweight/obesity among higher secondary school students was reported to be 8.1% with 2.3% obese and 5.8% overweight in Kaski district of Nepal [16]. However, there is a dearth of literature reporting the status of adolescent obesity from the rural section of Nepal. This study aims to find out the prevalence of obesity among adolescents of rural Nepal and its associated sociodemographic and behavioral factors. After childhood, adolescence is the next unrivaled stage of life for preventive and health promotion activities. Identification of high-risk behaviors among such adolescents will contribute in designing the appropriate intervention programs for encouraging them toward a healthy lifestyle.

## 2. Materials and Methods

### 2.1. Study Design

This study was an institution-based, cross-sectional analytical study designed to screen obesity among adolescent students and to explore factors associated with adolescence obesity in Gosaikunda rural municipality of Rasuwa district, Nepal. Data collection was performed from Sep 1 to 30 2022.

### 2.2. Study Settings and Participants

We conducted this study in secondary-level schools in Gosaikunda rural municipality of Rasuwa district, Nepal: Rasuwa Secondary School, Highland Secondary Boarding School, Namuna English Secondary Boarding School, and Shyame Wangphel Secondary School. All the students from grades 8, 9, and 10 present during the time of this study were enrolled in the study. Those students who were absent on the day of data collection were excluded from the study.

### 2.3. Study Sample

Sample size was calculated using openepi.com, an online statistical website. With a 4% absolute precision, 95% confidence interval, and an expected prevalence of adolescent obesity to be 11.1% (based on the prevalence of childhood obesity according to a previous study) [17], the sample size was calculated to be 237. Assuming a nonresponse rate of 12%, the total sample size was calculated to be about 267. Total enumerative sampling (consecutive sampling) was used for selecting both the study site and individual participants. The study was conducted in all four secondary schools in Gosaikunda rural municipality of Rasuwa district. All the students studying in grades 8, 9, and 10 in these schools were enrolled. We visited each school and took permission from the school administrations before conducting the study. The students and their parents were informed about the study objectives, and we took their consent for conducting the study.

### 2.4. Study Instruments

The Nepalese version of the standard Global School Health Survey (core questionnaire modules) was used to collect data on noncommunicable disease behavioral risk factors among the students. GSHS is a school health survey primarily conducted among students of the 13–17 years age group. In our study, we chose to enroll students from grades 8, 9, and 10. The GSHS survey questionnaire was developed by the World Health Organization (WHO) and the Centers for Disease Control and Prevention (CDC) for obtaining information from students to promote global school health and young health programs and policies. The GSHS questionnaire consists of ten core questionnaire modules which measure the behavior and protective factors associated with the leading causes of morbidity and mortality among children and adults. They include questions related to demography, alcohol use, dietary behaviors, drug use, hygiene, mental health, physical activity, protective factors, sexual behaviors, tobacco use, and violence and unintentional injury. The Nepalese version of the GSHS questionnaire has already been used in previous national school-based health surveys [18]. Permission was granted to use the Nepalese version of the GSHS questionnaire. Among the ten core questionnaire modules, questions related to demography, alcohol use, dietary behaviors, mental health, physical activity, and tobacco use were used in the present study to explore association with adolescent obesity.

### 2.5. Data Collection

After getting permission from the school administration and consent from students and parents, we distributed the self-administered questionnaires to all the students of grades 8, 9, and 10 of the selected schools, with instructions to fill up the questionnaires from coinvestigators and school teachers. Then, after collecting the filled-up questionnaires from the students, the height and weight of each student were measured and noted down in the same questionnaire. The height of the students was measured twice using a portable stadiometer and without shoes. It was measured in centimeters in one decimal figure, and the average of two readings was recorded. Weight was measured using digital weighing measures with minimal clothing and with shoes and taken in kilograms in two decimal figures. Two readings were taken, and the average of the two readings was recorded.

### 2.6. Outcome Variables

The prevalence of overweight/obesity was the main outcome of interest. The classification of the body mass index (BMI) was performed according to the Asia-Pacific classification of BMI. According to the classification, “overweight” was defined as having a BMI of 23–24.9 kilograms per meter square. “Obesity” was defined as having a BMI more than or equal to 25 kilograms per meter square.

### 2.7. Independent Variables

Sociodemographic variables of the participants included age, gender, grade of study (8, 9, or 10), ethnicity (categorized as Tamang and others), and religion (categorized as Buddhists and others).

#### 2.7.1. Dietary Characteristics

As evidenced in the previous study [19], vegetable and fruit intake among students was measured by a single self-reported question for each. The questions were “During the past 7 days, how many times did you eat fruit, such as banana and apple? ” and “During the past 7 days, how many times did you eat fruit, such as green leafy vegetables, cucumber, or carrot?” The options provided ranged from “I did not eat fruit during the past 7 days” to “4 or more times per day.” Just like the previous study, these variables were dichotomized into categories of eating fruits or vegetables daily and not eating fruits or vegetables daily [19]. Food security information was retrieved by the GSHS question “During the past 30 days, how often did you go hungry because there was not enough food in your home?” The options consisted of a 5-point Likert scale: never, rarely, sometimes, most of the times, and always. As evidenced in a previous study [20], food insecurity was indicated when the response was “sometimes,” “most of the time,” or “always.”

#### 2.7.2. Physical Activity

Physical activity was measured by a single self-reported question in the GSHS questionnaire module as used in a previous study [19]. The students were asked “During the past 7 days, on how many days were you physically active for a total of at least 60 minutes per day?” The provided options ranged from one to seven days. According to the WHO recommendation that there should be a minimum of 60 minutes of physical activity per day for five days and more drawing from past studies [19, 21], the participants were categorized into two groups. Those students who met the recommendation of engaging in physical activity of 5 days or more per week were categorized as “sufficiently physically active” and those who did not meet the recommendation were categorized as “physically inactive.” For assessing the attendance of students in physical education, the question was “During this school year, on how many days did you go to a physical education class each week?” The provided options ranged from 0 days to “5 days or more.” Students who attended physical education class at least 5 days per week were considered to have “regular attendance” [22]. We assessed sedentary behavior through the question “How much time do you spend during a typical or usual day sitting and watching television, playing computer games, talking with friends, or doing other seated activities such as surfing the Internet?” The provided responses were “less than 1 hour per day,” “1-2 hours per day,” “3-4 hours per day,” “5-6 hours per day,” “7-8 hours per day,” and “more than 8 hours per day.” Students spending 3 hours or more in these kinds of activities were considered to have “sedentary behavior” [22].

#### 2.7.3. Smoking and Alcohol Use

The questions asked for collecting information about current alcohol and tobacco use among adolescents were “During the past 30 days, on how many days did you have at least one drink containing alcohol?” and “During the past 30 days, on how many days did you smoke cigarettes?,” respectively. The responses for both of these questions ranged from “0 day” to “all 30 days.” Those students who drank alcohol/smoked cigarettes for 1 day or more were considered among “current alcohol use” and “current smoking” [23].

#### 2.7.4. Mental Health

For assessing loneliness and anxiety among adolescents, the questions were “During the past 12 months, how often did you feel lonely?” and “During the past 12 months, how often were you so worried about something that you could not sleep at night?,” respectively. The response options for both of these questions were “never,” “rarely,” “sometimes,” “most of the times,” and “always.” Adolescents responding “sometimes,” “most of the times,” and “always” for each question were considered as “feeling lonely” and having “anxiety” [24]. We assessed information about suicidal ideation among the adolescents through the question “During the past 12 months, did you ever seriously consider attempting suicide?” The response options were “yes” and “no,” and we categorized the adolescents based on these two responses.

### 2.8. Data Analysis

Data entry and analysis were performed using “SPSS” (Statistical Package for the Social Sciences) version 22. The dependent variable of the study was overweight/obesity based on the BMI of the participants. The independent variables were sociodemographic characteristics, dietary characteristics, physical activities, and sedentary behaviors of the students. The prevalence of adolescent obesity/overweight was reported as a proportion. The descriptive analysis of independent variables was reported as means, frequencies, and percentages. Logistic regression analysis was used to determine the association of independent variables and prevalence of overweight/obesity. Univariate and multivariate logistic regression analyses were performed to determine the relationship between dependent and independent variables. At first, variables were entered one at a time for univariate analysis, and unadjusted OR and 95% CI were computed with independent variables. Then, all the independent variables were entered at a time for multivariate analysis for adjusting the confounding effects, and adjusted OR and 95% CI were calculated. A *P* value of less than 0.05 was considered statistically significant.

### 2.9. Ethical Consideration

Ethical approval for the study was taken from the Nepal Health Research Council (NHRC) (Ref. No. 774). Written permission was taken from all school administrations. Before data collection, informed written consent was taken for all the students and from all the parents if the students were below 18 years. Written assent was taken from students below 18 years.

## 3. Results

### 3.1. Results of Descriptive Analysis

#### 3.1.1. Nutritional Status of Participants

**Figure 1:**
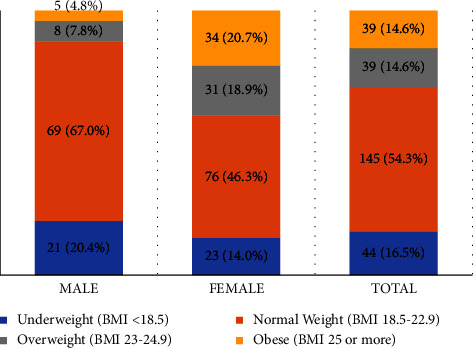
Classification of BMI among the respondents according to the Asia-Pacific classification. The figure shows the percentage of obese, overweight, and underweight participants in total and among males and females.

Out of total 267 participants, 14.6% (20.7% of females and 4.8% of males) were obese and 14.6% (18.9% of females and 7.8% of males) were overweight. Of total participants, 16.5% (14.0% of females and 20.4% of males) were underweight (Figure 1).

Table 1 shows the distribution of overweight/obesity by sociodemographic variables and other associated factors. Among 267 respondents, the majority (68.2%) belonged to the 15–17 years age group, and the mean age was 15.57 ± 1.43 years. 61.4% of the total respondents were female. Out of the total respondents, 29.6% studied in grade eight, 35.6% in grade nine, and 34.8% in grade ten. Of all the respondents, more than three-fourth (83.5%) belonged to the Tamang ethnicity and the majority (70%) were Buddhists. About three quarters of the participants (74.2%) were physically inactive, and only about 27.3% of them ate fruits daily.

In terms of gender, the prevalence of overweight/obesity was significantly higher among female participants (39.6%) than males (12.6%). In relation to the age group, the students of 15–17 years age had a significantly higher prevalence of overweight/obesity than age groups of 11–14 years and 18–21 years. Similarly, the number of obese/overweight students was significantly higher in grade ten (41.9%) than in grade nine (24.2%) and grade eight (20.2%). The prevalence of overweight/obesity was significantly higher among physically inactive students (32.8%) than in those who were sufficiently physically active (18.8%). The chi-square analysis showed that gender (*p* value <0.001), age (*p* value 0.01), grade of study (*p* value 0.003), and physical activity (*p* value 0.03) were significantly associated with overweight/obesity among adolescents.

### 3.2. Factors Associated with Adolescent Obesity/Overweight (Multivariate Analysis Results) (*n* = 267)

As shown in Table 2, gender, age, grade of study, and physical activity of the participants were significant predictors for adolescent obesity in univariate regression analysis. While in multivariate analysis after adjusting for the confounding factors, female gender (AOR: 5.2, 95% CI (2.5–10.9), *p* < 0.01) was the only significant independent predictor for obesity/overweight among adolescents.

## 4. Discussion

To determine the prevalence of obesity among adolescents in rural Nepal and its associated sociodemographic and behavioral variables, we performed a survey among 267 school students from Rasuwa, Nepal.

According to our research, 14.6% of participants (20.7% of females and 4.8% of males) are obese and 14.6% of participants (18.9% of females and 7.8% of males) are overweight. Therefore, overweight and obesity were prevalent at a rate of 29.2%. According to a survey conducted in Lalitpur Metropolitan, 18.6% of children were overweight and 7.1% were obese, making up the prevalence of overweight/obesity among children to be 25.7% [14]. According to another Indian survey, the frequency of obesity is 6.8% and the overweight population is roughly 17.1% [11]. As a result, our results are similar to those of studies conducted in India and another region of Nepal, which both indicate that a quarter of adolescents are obese and overweight.

According to the results of our research, female students were 5 times more likely than male students to be obese or overweight, which is similar to those of a study conducted in Kaski, Nepal [16]. This is in contrast to a study's result by Piryani S. et al. that indicated a greater prevalence in male than in female students [25]. The fact that the latter research was conducted in an urban setting as opposed to our study, which was conducted in a remote region of Nepal, could account for the discrepancy in the results. In our study setting which is a rural region of the country, adolescent boys are more active in sports and other outdoor activities compared to adolescent girls. Less physical activity among females may be one of the reasons for a higher prevalence of adolescent obesity among adolescent girls [26].

Furthermore, our study revealed a prevalence of underweight of 16.5% (14.0% of females and 20.4% of boys). This discovery falls outside of the focus of our research. However, we would like to stress that the rising prevalence of obesity and overweight does not rule out underweight; rather, as our research demonstrates, they coexist as an added burden.

In terms of physical activity, students who were physically inactive had a considerably greater prevalence of overweight/obesity (32.8%) than those who were adequately physically active (18.8%). Results from earlier research add support to this conclusion [14, 25]. Spending more than three hours a day seated while watching television, playing video games, or browsing the Internet not only keeps children from being physically inactive, but unhealthy food advertisements on TV and the Internet could also contribute to children becoming overweight or obese. [14, 25].

According to recent research, adolescents who ate fruit four times a week or less were three times more likely to be overweight than those who ate it more frequently [25]. Different research found that children's dietary preferences were an independent risk factor for being overweight [27]. Surprisingly, however, eating practices (consumption of fruits and/or vegetables) had no apparent effect on obesity in the present study.

Even after adjusting for possible confounding variables such as age, grade of study, and physical activity, the results of our research indicate that gender is a significant predictor of adolescent obesity. The likelihood of becoming obese or overweight during adolescents appears to be greater for females than for males [28, 29]. These findings are in accordance with a past study [16] that also found the female gender as a major risk factor for adolescent obesity. Gender disparities in BMI in adolescence may be linked to each gender's growth spurt and development of secondary sexual characteristics corresponding to the age range at which they have greater heights [28]. Puberty causes sexual dimorphism due to which males get more muscle-bone mass and females gain more fat mass [29]. Among females, early puberty has been associated with shorter height, higher BMI, and higher risk of obesity in adulthood [29]. However, it is crucial to take into account that other elements, such as age, grades of study, and physical activity, may also contribute to the development of obesity/overweight in adolescents. While these factors were significant predictors in our univariate analysis, they did not remain significant after adjusting for confounding factors in the multivariate analysis.

Our results have significant potential implications for the planning and implementation of preventive and therapeutic strategies for adolescent obesity. Particular attention may need to be paid to confronting gender-specific risk factors for adolescent obesity. However, it is also crucial to take into account additional variables that might increase the risk of becoming obese and to create comprehensive treatments that target these variables holistically.

## 5. Limitations

As the design of this study is cross-sectional, a causal link between the variables could not be determined. However, this study presents probable risk factors of adolescent obesity which can further be studied in the future by longitudinal studies. The study was conducted in secondary schools of a rural municipality of a rural district of Nepal, so the findings may not reflect the overall scenario of the country and may not be generalizable for adolescents in urban regions. We used the GSHS questionnaire to obtain information from the participants about the associated risk factors in which we used a single-item question to measure various characteristics such as “physical activity,” “attendance in physical education,” “fruit consumption,” “vegetable consumption,” “sedentary behavior,” and “food insecurity.” Each of these characteristics could be measured more comprehensively using elaborate tools which offer better insights and assessment of risk behaviors. The use of a self-reported questionnaire for data collection could have led to recall biases, so the findings need to be interpreted cautiously. As the behavioral findings were based on self-reported subjective measurements, social desirability bias was likely to occur which could have led to under reporting of undesirable variables, especially such as alcohol consumption and smoking.

## 6. Conclusions

More than one quarter of school going adolescents in Rasuwa district of Nepal were either overweight or obese. The prevalence of adolescent overweight/obesity was significantly more among adolescent females than males. This signifies the need to address gender-specific risk factors for adolescent obesity. School-based health intervention programs to promote healthy lifestyles among adolescents for maintaining healthy weight status are suggested. Further comprehensive studies with more specific analysis and objective measurements to determine the association between various risk behaviors and adolescent overweight/obesity among Nepalese adolescents are highly recommended.

## Figures and Tables

**Table 1:** Distribution of the sample according to obesity/overweight.

Variables	Frequency (*n* = 267)	Percentage	Percentage of obese/overweight	95% CI	*p* value
Sex					
Female	164	61.4	39.6	32.3–47.3	<0.01
Male	103	38.6	12.6	7.5–20.4	
Age					
11–14 years	66	24.7	15.1	8.4–25.7	0.01
15–17 years	182	68.2	34.6	28.1–41.8	
18–21 years	19	7.1	26.3	11.8–48.8	
Grade of study					
Eight	79	29.6	20.2	12.9–30.4	<0.01
Nine	95	35.6	24.2	16.7–33.7	
Ten	93	34.8	41.9	32.4–52.1	
Caste					
Tamang	223	83.5	28.7	23.2–35.0	0.678
Others	44	16.5	31.8	20.0–46.6	
Religion					
Buddhists	187	70.0	26.7	20.9–33.5	0.174
Others	80	30.0	35.0	25.4–45.9	
Physical activity					
Physically active	69	25.8	18.8	11.3–29.6	0.03
Physically inactive	198	74.2	32.8	26.7–39.6	
Physical education					
Regular attendance	81	30.3	21.0	13.5–31.1	0.051
Irregular attendance	186	69.7	32.8	26.5–39.8	
Food insecurity					
Yes	47	17.6	29.8	18.7–44.0	0.92
No	220	82.4	29.1	23.5–35.4	
Ate fruits daily					
Yes	73	27.3	24.7	16.2–35.6	0.315
No	194	72.7	30.9	24.8–37.7	
Ate vegetables daily					
Yes	136	50.9	26.5	19.8–34.5	0.315
No	131	49.1	32.1	24.7–40.5	
Current alcohol use					
Yes	12	4.5	41.7	19.3–68.1	0.332
No	255	95.5	28.6	23.4–34.5	
Current smoking					
Yes	8	3.0	0.0		0.06
No	259	97.0	30.1	24.9–36.0	
Sedentary behavior					
Yes	50	18.7	22.0	12.8–35.2	0.213
No	217	81.3	30.9	25.1–37.3	
Feeling lonely					
Yes	144	53.9	33.3	26.2–41.2	0.109
No	123	46.1	24.4	17.6–32.3	
Anxiety					
Yes	100	37.5	32.0	23.7–41.7	0.438
No	167	62.5	27.5	21.3–34.8	
Suicidal ideation					
Yes	16	6.0	25.0	10.2–49.5	0.71
No	251	94.0	29.5	24.2–35.4	

**Table 2:** Multivariate logistic regression analysis showing associated factors with obesity/overweight among adolescents.

Independent variables	COR (95% CI)	*p* value	AOR (95% CI)	*p* value
Sex				
Male	Ref	<0.01	Ref	<0.01
Female	4.5 (2.3–8.8)		5.2 (2.5–10.9)	
Age				
11–14 years	Ref		Ref	
15–17 years	3.0 (1.4–6.2)	0.015	2.4 (1.0–5.7)	0.11
18–21 years	2.0 (0.6–6.8)		1.6 (0.4–6.6)	
Grade of study				
Eight	Ref		Ref	
Nine	1.3 (0.6–2.6)	<0.01	0.9 (0.4–2.0)	0.19
Ten	2.8 (1.4–5.6)		1.7 (0.7–4.1)	
Caste				
Tamang	Ref	0.68	Ref	0.49
Others	1.2 (0.6–2.30		1.3 (0.6–3.1)	
Religion				
Buddhists	Ref	0.17	Ref	0.73
Others	1.5 (0.8–2.6)		0.9 (0.4–1.8)	
Physical activity				
Physically active	Ref	0.03	Ref	0.42
Physically inactive	2.1 (1.1–4.1)		1.4 (0.6–3.1)	
Physical education				
Regular attendance	Ref	0.053	Ref	0.38
Irregular attendance	1.8 (1.0–3.4)		1.4 (0.7–2.9)	
Food insecurity				
Yes	Ref	0.92	Ref	0.79
No	1.0 (0.5–1.9)		0.9 (0.4–2.0)	
Ate fruits daily				
Yes	Ref	0.32	Ref	0.98
No	1.4 (0.7–2.5)		1.0 (0.5–2.0)	
Ate vegetables daily				
Yes	Ref	0.32	Ref	0.79
No	1.3 (0.8–2.2)		1.1 (0.6–2.0)	
Current alcohol use				
Yes	Ref	0.34	Ref	0.19
No	0.6 (0.2–1.8)		0.4 (0.8–1.6)	
Sedentary behavior				
Yes	Ref	0.22	Ref	0.84
No	1.6 (0.8–3.3)		1.1 (0.5–2.6)	
Feeling lonely				
Yes	Ref	0.11	Ref	0.09
No	0.6 (0.4–1.1)		0.6 (0.3–1.1)	
Anxiety				
Yes	Ref	0.44	Ref	0.90
No	0.8 (0.5–1.4)		1.0 (0.5–1.9)	
Suicidal ideation				
Yes	Ref	0.70	Ref	0.19
No	1.2 (0.4–4.0)		2.5 (0.6–10.0)	

## Data Availability

The raw data under identification policy will be provided on request through e-mail to the corresponding author.
